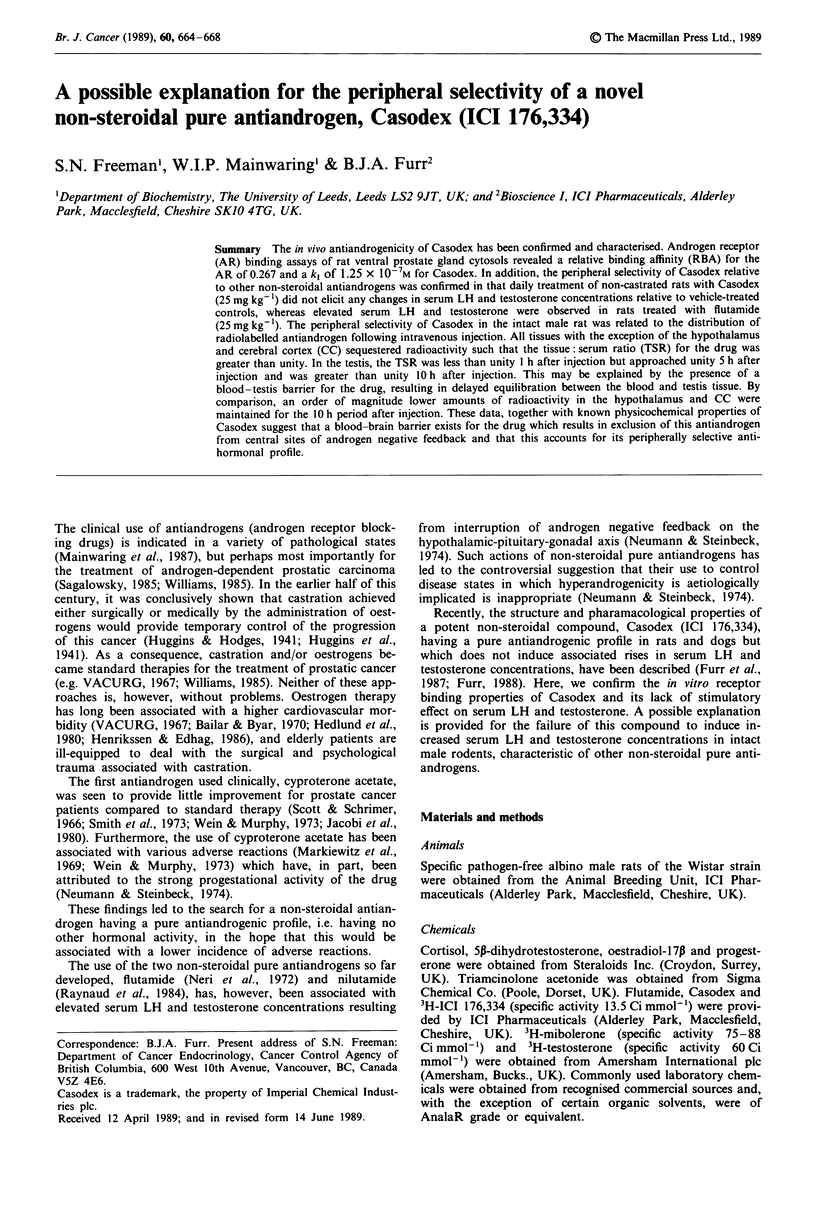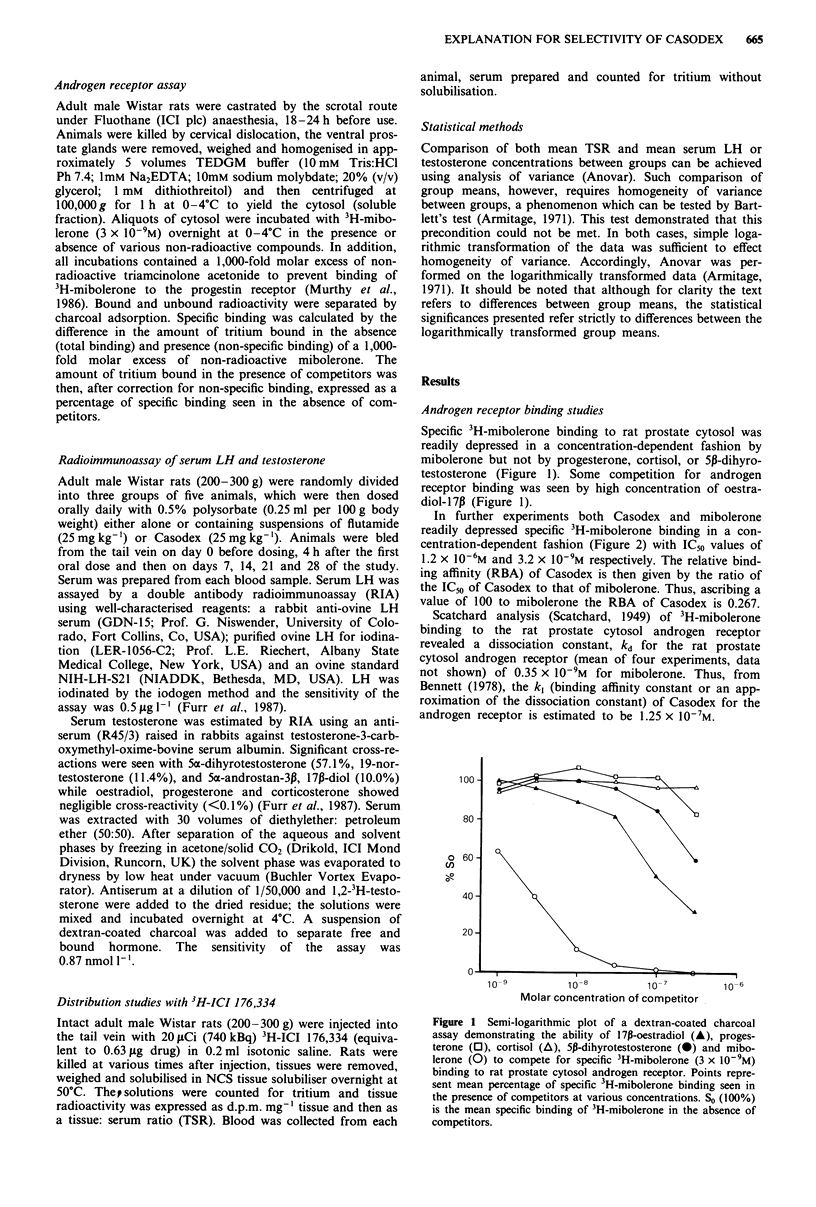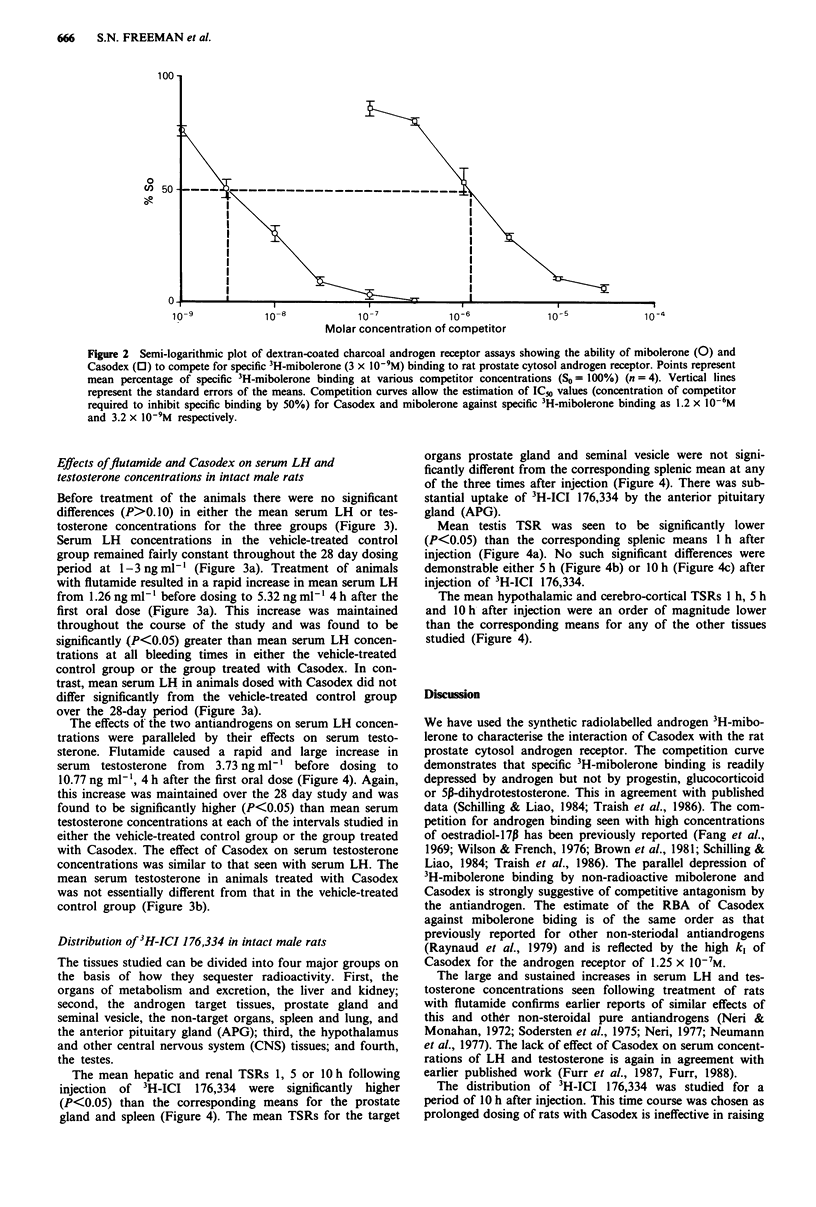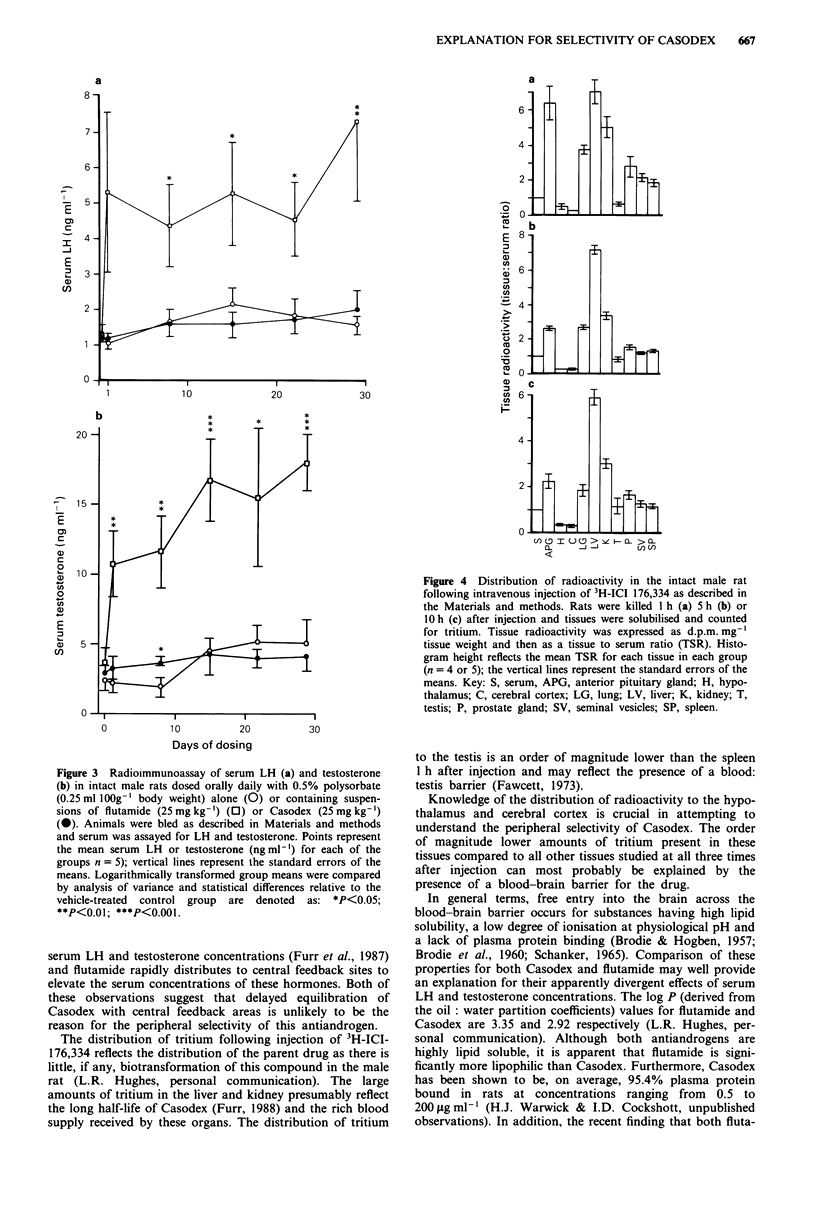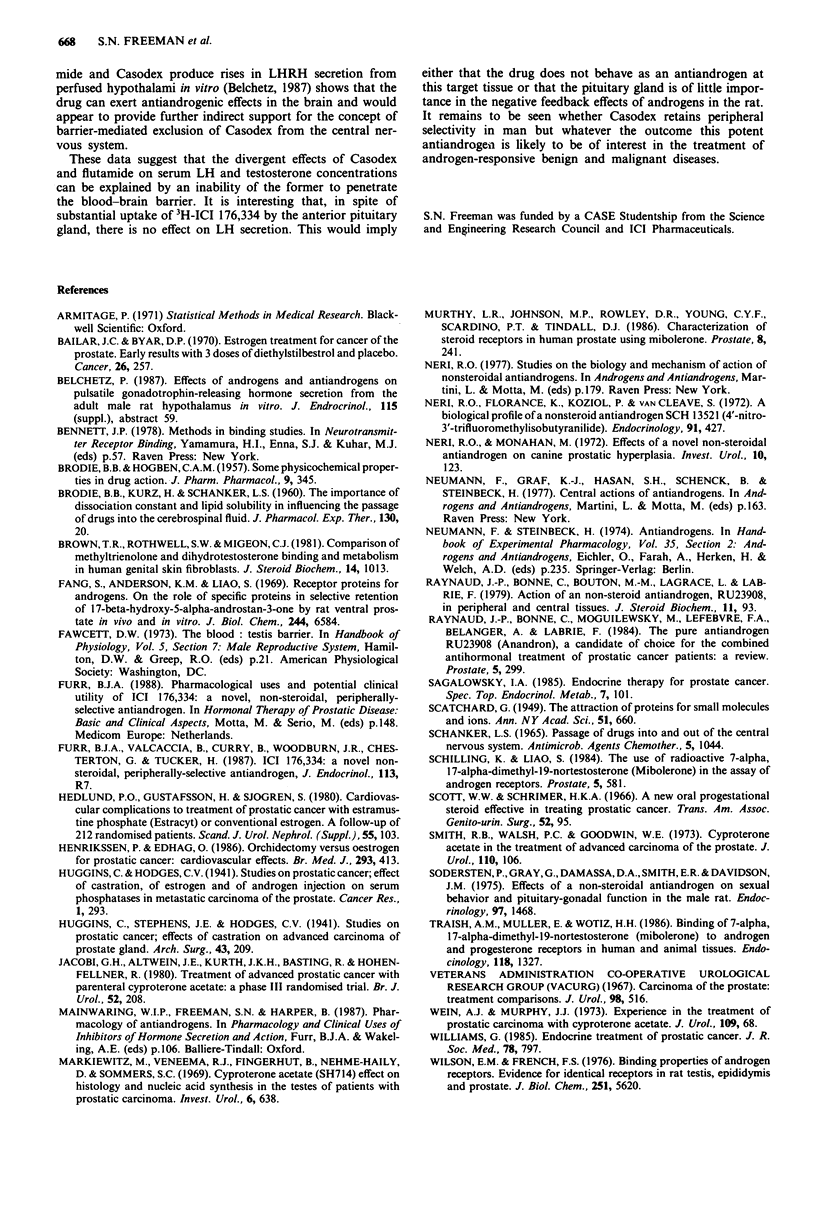# A possible explanation for the peripheral selectivity of a novel non-steroidal pure antiandrogen, Casodex (ICI 176,334).

**DOI:** 10.1038/bjc.1989.336

**Published:** 1989-11

**Authors:** S. N. Freeman, W. I. Mainwaring, B. J. Furr

**Affiliations:** Department of Biochemistry, University of Leeds, UK.

## Abstract

The in vivo antiandrogenicity of Casodex has been confirmed and characterised. Androgen receptor (AR) binding assays of rat ventral prostate gland cytosols revealed a relative binding affinity (RBA) for the AR of 0.267 and a k1 of 1.25 x 10(-7) M for Casodex. In addition, the peripheral selectivity of Casodex relative to other non-steroidal antiandrogens was confirmed in that daily treatment of non-castrated rats with Casodex (25 mg kg-1) did not elicit any changes in serum LH and testosterone concentrations relative to vehicle-treated controls, whereas elevated serum LH and testosterone were observed in rats treated with flutamide (25 mg kg-1). The peripheral selectivity of Casodex in the intact male rat was related to the distribution of radiolabelled antiandrogen following intravenous injection. All tissues with the exception of the hypothalamus and cerebral cortex (CC) sequestered radioactivity such that the tissue:serum ratio (TSR) for the drug was greater than unity. In the testis, the TSR was less than unity 1 h after injection but approached unity 5 h after injection and was greater than unity 10 h after injection. This may be explained by the presence of a blood-testis barrier for the drug, resulting in delayed equilibration between the blood and testis tissue. By comparison, an order of magnitude lower amounts of radioactivity in the hypothalamus and CC were maintained for the 10 h period after injection. These data, together with known physicochemical properties of Casodex suggest that a blood-brain barrier exists for the drug which results in exclusion of this antiandrogen from central sites of androgen negative feedback and that this accounts for its peripherally selective antihormonal profile.


Br. J. Cancer (1989), 60, 664-668 © The Macmillan Press Ltd., 1989

### A possible explanation for the peripheral selectivity of a novel non-steroidal pure antiandrogen, Casodex (ICI 176,334)

S.N. Freeman[1], W.I.P. Mainwaring[1] & B.J.A. Furr[2]

[1]*Department of Biochemistry, The University of Leeds, Leeds LS2 9JT, UK; and* [2]*Bioscience I, ICI Pharmaceuticals, Alderley Park, Macclesfield, Cheshire SK10 4TG, UK.*

**Summary** The *in vivo* antiandrogenicity of Casodex has been confirmed and characterised. Androgen receptor (AR) binding assays of rat ventral prostate gland cytosols revealed a relative binding affinity (RBA) for the AR of 0.267 and a $k_I$ of $1.25 \times 10^{-7}$M for Casodex. In addition, the peripheral selectivity of Casodex relative to other non-steroidal antiandrogens was confirmed in that daily treatment of non-castrated rats with Casodex (25 mg $kg^{-1}$) did not elicit any changes in serum LH and testosterone concentrations relative to vehicle-treated controls, whereas elevated serum LH and testosterone were observed in rats treated with flutamide (25 mg $kg^{-1}$). The peripheral selectivity of Casodex in the intact male rat was related to the distribution of radiolabelled antiandrogen following intravenous injection. All tissues with the exception of the hypothalamus and cerebral cortex (CC) sequestered radioactivity such that the tissue : serum ratio (TSR) for the drug was greater than unity. In the testis, the TSR was less than unity 1 h after injection but approached unity 5 h after injection and was greater than unity 10 h after injection. This may be explained by the presence of a blood-testis barrier for the drug, resulting in delayed equilibration between the blood and testis tissue. By comparison, an order of magnitude lower amounts of radioactivity in the hypothalamus and CC were maintained for the 10 h period after injection. These data, together with known physicochemical properties of Casodex suggest that a blood-brain barrier exists for the drug which results in exclusion of this antiandrogen from central sites of androgen negative feedback and that this accounts for its peripherally selective anti-hormonal profile.

The clinical use of antiandrogens (androgen receptor blocking drugs) is indicated in a variety of pathological states (Mainwaring *et al.*, 1987), but perhaps most importantly for the treatment of androgen-dependent prostatic carcinoma (Sagalowsky, 1985; Williams, 1985). In the earlier half of this century, it was conclusively shown that castration achieved either surgically or medically by the administration of oestrogens would provide temporary control of the progression of this cancer (Huggins & Hodges, 1941; Huggins *et al.*, 1941). As a consequence, castration and/or oestrogens became standard therapies for the treatment of prostatic cancer (e.g. VACURG, 1967; Williams, 1985). Neither of these approaches is, however, without problems. Oestrogen therapy has long been associated with a higher cardiovascular morbidity (VACURG, 1967; Bailar & Byar, 1970; Hedlund *et al.*, 1980; Henrikssen & Edhag, 1986), and elderly patients are ill-equipped to deal with the surgical and psychological trauma associated with castration.

The first antiandrogen used clinically, cyproterone acetate, was seen to provide little improvement for prostate cancer patients compared to standard therapy (Scott & Schrimer, 1966; Smith *et al.*, 1973; Wein & Murphy, 1973; Jacobi *et al.*, 1980). Furthermore, the use of cyproterone acetate has been associated with various adverse reactions (Markiewitz *et al.*, 1969; Wein & Murphy, 1973) which have, in part, been attributed to the strong progestational activity of the drug (Neumann & Steinbeck, 1974).

These findings led to the search for a non-steroidal antiandrogen having a pure antiandrogenic profile, i.e. having no other hormonal activity, in the hope that this would be associated with a lower incidence of adverse reactions.

The use of the two non-steroidal pure antiandrogens so far developed, flutamide (Neri *et al.*, 1972) and nilutamide (Raynaud *et al.*, 1984), has, however, been associated with elevated serum LH and testosterone concentrations resulting from interruption of androgen negative feedback on the hypothalamic-pituitary-gonadal axis (Neumann & Steinbeck, 1974). Such actions of non-steroidal pure antiandrogens has led to the controversial suggestion that their use to control disease states in which hyperandrogenicity is aetiologically implicated is inappropriate (Neumann & Steinbeck, 1974).

Recently, the structure and pharamacological properties of a potent non-steroidal compound, Casodex (ICI 176,334), having a pure antiandrogenic profile in rats and dogs but which does not induce associated rises in serum LH and testosterone concentrations, have been described (Furr *et al.*, 1987; Furr, 1988). Here, we confirm the *in vitro* receptor binding properties of Casodex and its lack of stimulatory effect on serum LH and testosterone. A possible explanation is provided for the failure of this compound to induce increased serum LH and testosterone concentrations in intact male rodents, characteristic of other non-steroidal pure antiandrogens.

### Materials and methods

### *Animals*

Specific pathogen-free albino male rats of the Wistar strain were obtained from the Animal Breeding Unit, ICI Pharmaceuticals (Alderley Park, Macclesfield, Cheshire, UK).

### *Chemicals*

Cortisol, 5β-dihydrotestosterone, oestradiol-17β and progesterone were obtained from Steraloids Inc. (Croydon, Surrey, UK). Triamcinolone acetonide was obtained from Sigma Chemical Co. (Poole, Dorset, UK). Flutamide, Casodex and $^3$H-ICI 176,334 (specific activity 13.5 Ci $mmol^{-1}$) were provided by ICI Pharmaceuticals (Alderley Park, Macclesfield, Cheshire, UK). $^3$H-mibolerone (specific activity 75-88 Ci $mmol^{-1}$) and $^3$H-testosterone (specific activity 60 Ci $mmol^{-1}$) were obtained from Amersham International plc (Amersham, Bucks., UK). Commonly used laboratory chemicals were obtained from recognised commercial sources and, with the exception of certain organic solvents, were of AnalaR grade or equivalent.

Correspondence: B.J.A. Furr. Present address of S.N. Freeman: Department of Cancer Endocrinology, Cancer Control Agency of British Columbia, 600 West 10th Avenue, Vancouver, BC, Canada V5Z 4E6.
Casodex is a trademark, the property of Imperial Chemical Industries plc.
Received 12 April 1989; and in revised form 14 June 1989.

*Androgen receptor assay*

Adult male Wistar rats were castrated by the scrotal route under Fluothane (ICI plc) anaesthesia, 18–24 h before use. Animals were killed by cervical dislocation, the ventral prostate glands were removed, weighed and homogenised in approximately 5 volumes TEDGM buffer (10 mM Tris:HCl Ph 7.4; 1mM $Na_2EDTA$; 10mM sodium molybdate; 20% (v/v) glycerol; 1 mM dithiothreitol) and then centrifuged at 100,000 *g* for 1 h at 0–4°C to yield the cytosol (soluble fraction). Aliquots of cytosol were incubated with $^3$H-mibolerone ($3 \times 10^{-9}$M) overnight at 0–4°C in the presence or absence of various non-radioactive compounds. In addition, all incubations contained a 1,000-fold molar excess of non-radioactive triamcinolone acetonide to prevent binding of $^3$H-mibolerone to the progestin receptor (Murthy *et al.*, 1986). Bound and unbound radioactivity were separated by charcoal adsorption. Specific binding was calculated by the difference in the amount of tritium bound in the absence (total binding) and presence (non-specific binding) of a 1,000-fold molar excess of non-radioactive mibolerone. The amount of tritium bound in the presence of competitors was then, after correction for non-specific binding, expressed as a percentage of specific binding seen in the absence of competitors.

*Radioimmunoassay of serum LH and testosterone*

Adult male Wistar rats (200–300 g) were randomly divided into three groups of five animals, which were then dosed orally daily with 0.5% polysorbate (0.25 ml per 100 g body weight) either alone or containing suspensions of flutamide (25 mg kg$^{-1}$) or Casodex (25 mg kg$^{-1}$). Animals were bled from the tail vein on day 0 before dosing, 4 h after the first oral dose and then on days 7, 14, 21 and 28 of the study. Serum was prepared from each blood sample. Serum LH was assayed by a double antibody radioimmunoassay (RIA) using well-characterised reagents: a rabbit anti-ovine LH serum (GDN-15; Prof. G. Niswender, University of Colorado, Fort Collins, Co, USA); purified ovine LH for iodination (LER-1056-C2; Prof. L.E. Riechert, Albany State Medical College, New York, USA) and an ovine standard NIH-LH-S21 (NIADDK, Bethesda, MD, USA). LH was iodinated by the iodogen method and the sensitivity of the assay was 0.5 $\mu$g l$^{-1}$ (Furr *et al.*, 1987).

Serum testosterone was estimated by RIA using an antiserum (R45/3) raised in rabbits against testosterone-3-carboxymethyl-oxime-bovine serum albumin. Significant cross-reactions were seen with 5α-dihyrotestosterone (57.1%, 19-nortestosterone (11.4%), and 5α-androstan-3β, 17β-diol (10.0%) while oestradiol, progesterone and corticosterone showed negligible cross-reactivity (<0.1%) (Furr *et al.*, 1987). Serum was extracted with 30 volumes of diethylether: petroleum ether (50:50). After separation of the aqueous and solvent phases by freezing in acetone/solid $CO_2$ (Drikold, ICI Mond Division, Runcorn, UK) the solvent phase was evaporated to dryness by low heat under vacuum (Buchler Vortex Evaporator). Antiserum at a dilution of 1/50,000 and 1,2-$^3$H-testosterone were added to the dried residue; the solutions were mixed and incubated overnight at 4°C. A suspension of dextran-coated charcoal was added to separate free and bound hormone. The sensitivity of the assay was 0.87 nmol l$^{-1}$.

*Distribution studies with $^3$H-ICI 176,334*

Intact adult male Wistar rats (200–300 g) were injected into the tail vein with 20 $\mu$Ci (740 kBq) $^3$H-ICI 176,334 (equivalent to 0.63 $\mu$g drug) in 0.2 ml isotonic saline. Rats were killed at various times after injection, tissues were removed, weighed and solubilised in NCS tissue solubiliser overnight at 50°C. The solutions were counted for tritium and tissue radioactivity was expressed as d.p.m. mg$^{-1}$ tissue and then as a tissue: serum ratio (TSR). Blood was collected from each animal, serum prepared and counted for tritium without solubilisation.

*Statistical methods*

Comparison of both mean TSR and mean serum LH or testosterone concentrations between groups can be achieved using analysis of variance (Anovar). Such comparison of group means, however, requires homogeneity of variance between groups, a phenomenon which can be tested by Bartlett's test (Armitage, 1971). This test demonstrated that this precondition could not be met. In both cases, simple logarithmic transformation of the data was sufficient to effect homogeneity of variance. Accordingly, Anovar was performed on the logarithmically transformed data (Armitage, 1971). It should be noted that although for clarity the text refers to differences between group means, the statistical significances presented refer strictly to differences between the logarithmically transformed group means.

### Results

*Androgen receptor binding studies*

Specific $^3$H-mibolerone binding to rat prostate cytosol was readily depressed in a concentration-dependent fashion by mibolerone but not by progesterone, cortisol, or 5β-dihyrotestosterone (Figure 1). Some competition for androgen receptor binding was seen by high concentration of oestradiol-17β (Figure 1).

In further experiments both Casodex and mibolerone readily depressed specific $^3$H-mibolerone binding in a concentration-dependent fashion (Figure 2) with $IC_{50}$ values of $1.2 \times 10^{-6}$M and $3.2 \times 10^{-9}$M respectively. The relative binding affinity (RBA) of Casodex is then given by the ratio of the $IC_{50}$ of Casodex to that of mibolerone. Thus, ascribing a value of 100 to mibolerone the RBA of Casodex is 0.267.

Scatchard analysis (Scatchard, 1949) of $^3$H-mibolerone binding to the rat prostate cytosol androgen receptor revealed a dissociation constant, $k_d$ for the rat prostate cytosol androgen receptor (mean of four experiments, data not shown) of $0.35 \times 10^{-9}$M for mibolerone. Thus, from Bennett (1978), the $k_I$ (binding affinity constant or an approximation of the dissociation constant) of Casodex for the androgen receptor is estimated to be $1.25 \times 10^{-7}$M.

**Figure 1** Semi-logarithmic plot of a dextran-coated charcoal assay demonstrating the ability of 17β-oestradiol (▲), progesterone (□), cortisol (△), 5β-dihyrotestosterone (●) and mibolerone (○) to compete for specific $^3$H-mibolerone ($3 \times 10^{-9}$M) binding to rat prostate cytosol androgen receptor. Points represent mean percentage of specific $^3$H-mibolerone binding seen in the presence of competitors at various concentrations. $S_0$ (100%) is the mean specific binding of $^3$H-mibolerone in the absence of competitors.

**Figure 2** Semi-logarithmic plot of dextran-coated charcoal androgen receptor assays showing the ability of mibolerone (O) and Casodex (□) to compete for specific $^3$H-mibolerone ($3 \times 10^{-9}$M) binding to rat prostate cytosol androgen receptor. Points represent mean percentage of specific $^3$H-mibolerone binding at various competitor concentrations ($S_0 = 100\%$) ($n = 4$). Vertical lines represent the standard errors of the means. Competition curves allow the estimation of $IC_{50}$ values (concentration of competitor required to inhibit specific binding by 50%) for Casodex and mibolerone against specific $^3$H-mibolerone binding as $1.2 \times 10^{-6}$M and $3.2 \times 10^{-9}$M respectively.

### *Effects of flutamide and Casodex on serum LH and testosterone concentrations in intact male rats*

Before treatment of the animals there were no significant differences ($P>0.10$) in either the mean serum LH or testosterone concentrations for the three groups (Figure 3). Serum LH concentrations in the vehicle-treated control group remained fairly constant throughout the 28 day dosing period at 1–3 ng $ml^{-1}$ (Figure 3a). Treatment of animals with flutamide resulted in a rapid increase in mean serum LH from 1.26 ng $ml^{-1}$ before dosing to 5.32 ng $ml^{-1}$ 4 h after the first oral dose (Figure 3a). This increase was maintained throughout the course of the study and was found to be significantly ($P<0.05$) greater than mean serum LH concentrations at all bleeding times in either the vehicle-treated control group or the group treated with Casodex. In contrast, mean serum LH in animals dosed with Casodex did not differ significantly from the vehicle-treated control group over the 28-day period (Figure 3a).

The effects of the two antiandrogens on serum LH concentrations were paralleled by their effects on serum testosterone. Flutamide caused a rapid and large increase in serum testosterone from 3.73 ng $ml^{-1}$ before dosing to 10.77 ng $ml^{-1}$, 4 h after the first oral dose (Figure 4). Again, this increase was maintained over the 28 day study and was found to be significantly higher ($P<0.05$) than mean serum testosterone concentrations at each of the intervals studied in either the vehicle-treated control group or the group treated with Casodex. The effect of Casodex on serum testosterone concentrations was similar to that seen with serum LH. The mean serum testosterone in animals treated with Casodex was not essentially different from that in the vehicle-treated control group (Figure 3b).

### *Distribution of $^3$H-ICI 176,334 in intact male rats*

The tissues studied can be divided into four major groups on the basis of how they sequester radioactivity. First, the organs of metabolism and excretion, the liver and kidney; second, the androgen target tissues, prostate gland and seminal vesicle, the non-target organs, spleen and lung, and the anterior pituitary gland (APG); third, the hypothalamus and other central nervous system (CNS) tissues; and fourth, the testes.

The mean hepatic and renal TSRs 1, 5 or 10 h following injection of $^3$H-ICI 176,334 were significantly higher ($P<0.05$) than the corresponding means for the prostate gland and spleen (Figure 4). The mean TSRs for the target organs prostate gland and seminal vesicle were not significantly different from the corresponding splenic mean at any of the three times after injection (Figure 4). There was substantial uptake of $^3$H-ICI 176,334 by the anterior pituitary gland (APG).

Mean testis TSR was seen to be significantly lower ($P<0.05$) than the corresponding splenic means 1 h after injection (Figure 4a). No such significant differences were demonstrable either 5 h (Figure 4b) or 10 h (Figure 4c) after injection of $^3$H-ICI 176,334.

The mean hypothalamic and cerebro-cortical TSRs 1 h, 5 h and 10 h after injection were an order of magnitude lower than the corresponding means for any of the other tissues studied (Figure 4).

### Discussion

We have used the synthetic radiolabelled androgen $^3$H-mibolerone to characterise the interaction of Casodex with the rat prostate cytosol androgen receptor. The competition curve demonstrates that specific $^3$H-mibolerone binding is readily depressed by androgen but not by progestin, glucocorticoid or 5β-dihydrotestosterone. This in agreement with published data (Schilling & Liao, 1984; Traish *et al.*, 1986). The competition for androgen binding seen with high concentrations of oestradiol-17β has been previously reported (Fang *et al.*, 1969; Wilson & French, 1976; Brown *et al.*, 1981; Schilling & Liao, 1984; Traish *et al.*, 1986). The parallel depression of $^3$H-mibolerone binding by non-radioactive mibolerone and Casodex is strongly suggestive of competitive antagonism by the antiandrogen. The estimate of the RBA of Casodex against mibolerone biding is of the same order as that previously reported for other non-steriodal antiandrogens (Raynaud *et al.*, 1979) and is reflected by the high $k_i$ of Casodex for the androgen receptor of $1.25 \times 10^{-7}$M.

The large and sustained increases in serum LH and testosterone concentrations seen following treatment of rats with flutamide confirms earlier reports of similar effects of this and other non-steroidal pure antiandrogens (Neri & Monahan, 1972; Sodersten *et al.*, 1975; Neri, 1977; Neumann *et al.*, 1977). The lack of effect of Casodex on serum concentrations of LH and testosterone is again in agreement with earlier published work (Furr *et al.*, 1987, Furr, 1988).

The distribution of $^3$H-ICI 176,334 was studied for a period of 10 h after injection. This time course was chosen as prolonged dosing of rats with Casodex is ineffective in raising

**Figure 3** Radioimmunoassay of serum LH (**a**) and testosterone (**b**) in intact male rats dosed orally daily with 0.5% polysorbate (0.25 ml $100g^{-1}$ body weight) alone (○) or containing suspensions of flutamide ($25\,mg\,kg^{-1}$) (□) or Casodex ($25\,mg\,kg^{-1}$) (●). Animals were bled as described in Materials and methods and serum was assayed for LH and testosterone. Points represent the mean serum LH or testosterone ($ng\,ml^{-1}$) for each of the groups $n = 5$); vertical lines represent the standard errors of the means. Logarithmically transformed group means were compared by analysis of variance and statistical differences relative to the vehicle-treated control group are denoted as: $^{*}P<0.05$; $^{**}P<0.01$; $^{***}P<0.001$.

**Figure 4** Distribution of radioactivity in the intact male rat following intravenous injection of $^{3}$H-ICI 176,334 as described in the Materials and methods. Rats were killed 1 h (**a**) 5 h (**b**) or 10 h (**c**) after injection and tissues were solubilised and counted for tritium. Tissue radioactivity was expressed as d.p.m. $mg^{-1}$ tissue weight and then as a tissue to serum ratio (TSR). Histogram height reflects the mean TSR for each tissue in each group ($n = 4$ or 5); the vertical lines represent the standard errors of the means. Key: S, serum, APG, anterior pituitary gland; H, hypothalamus; C, cerebral cortex; LG, lung; LV, liver; K, kidney; T, testis; P, prostate gland; SV, seminal vesicles; SP, spleen.

serum LH and testosterone concentrations (Furr *et al.*, 1987) and flutamide rapidly distributes to central feedback sites to elevate the serum concentrations of these hormones. Both of these observations suggest that delayed equilibration of Casodex with central feedback areas is unlikely to be the reason for the peripheral selectivity of this antiandrogen.

The distribution of tritium following injection of $^{3}$H-ICI-176,334 reflects the distribution of the parent drug as there is little, if any, biotransformation of this compound in the male rat (L.R. Hughes, personal communication). The large amounts of tritium in the liver and kidney presumably reflect the long half-life of Casodex (Furr, 1988) and the rich blood supply received by these organs. The distribution of tritium to the testis is an order of magnitude lower than the spleen 1 h after injection and may reflect the presence of a blood: testis barrier (Fawcett, 1973).

Knowledge of the distribution of radioactivity to the hypothalamus and cerebral cortex is crucial in attempting to understand the peripheral selectivity of Casodex. The order of magnitude lower amounts of tritium present in these tissues compared to all other tissues studied at all three times after injection can most probably be explained by the presence of a blood–brain barrier for the drug.

In general terms, free entry into the brain across the blood–brain barrier occurs for substances having high lipid solubility, a low degree of ionisation at physiological pH and a lack of plasma protein binding (Brodie & Hogben, 1957; Brodie *et al.*, 1960; Schanker, 1965). Comparison of these properties for both Casodex and flutamide may well provide an explanation for their apparently divergent effects of serum LH and testosterone concentrations. The log *P* (derived from the oil : water partition coefficients) values for flutamide and Casodex are 3.35 and 2.92 respectively (L.R. Hughes, personal communication). Although both antiandrogens are highly lipid soluble, it is apparent that flutamide is significantly more lipophilic than Casodex. Furthermore, Casodex has been shown to be, on average, 95.4% plasma protein bound in rats at concentrations ranging from 0.5 to $200\,\mu g\,ml^{-1}$ (H.J. Warwick & I.D. Cockshott, unpublished observations). In addition, the recent finding that both flutamide and Casodex produce rises in LHRH secretion from perfused hypothalami *in vitro* (Belchetz, 1987) shows that the drug can exert antiandrogenic effects in the brain and would appear to provide further indirect support for the concept of barrier-mediated exclusion of Casodex from the central nervous system.

These data suggest that the divergent effects of Casodex and flutamide on serum LH and testosterone concentrations can be explained by an inability of the former to penetrate the blood–brain barrier. It is interesting that, in spite of substantial uptake of $^3$H-ICI 176,334 by the anterior pituitary gland, there is no effect on LH secretion. This would imply either that the drug does not behave as an antiandrogen at this target tissue or that the pituitary gland is of little importance in the negative feedback effects of androgens in the rat. It remains to be seen whether Casodex retains peripheral selectivity in man but whatever the outcome this potent antiandrogen is likely to be of interest in the treatment of androgen-responsive benign and malignant diseases.

S.N. Freeman was funded by a CASE Studentship from the Science and Engineering Research Council and ICI Pharmaceuticals.

### References

ARMITAGE, P. (1971) *Statistical Methods in Medical Research.* Blackwell Scientific: Oxford.

BAILAR, J.C. & BYAR, D.P. (1970). Estrogen treatment for cancer of the prostate. Early results with 3 doses of diethylstilbestrol and placebo. *Cancer*, **26**, 257.

BELCHETZ, P. (1987). Effects of androgens and antiandrogens on pulsatile gonadotrophin-releasing hormone secretion from the adult male rat hypothalamus *in vitro*. *J. Endocrinol.*, **115** (suppl.), abstract 59.

BENNETT, J.P. (1978). Methods in binding studies. In *Neurotransmitter Receptor Binding*, Yamamura, H.I., Enna, S.J. & Kuhar, M.J. (eds) p.57. Raven Press: New York.

BRODIE, B.B. & HOGBEN, C.A.M. (1957). Some physicochemical properties in drug action. *J. Pharm. Pharmacol.*, **9**, 345.

BRODIE, B.B., KURZ, H. & SCHANKER, L.S. (1960). The importance of dissociation constant and lipid solubility in influencing the passage of drugs into the cerebrospinal fluid. *J. Pharmacol. Exp. Ther.*, **130**, 20.

BROWN, T.R., ROTHWELL, S.W. & MIGEON, C.J. (1981). Comparison of methyltrienolone and dihydrotestosterone binding and metabolism in human genital skin fibroblasts. *J. Steroid Biochem.*, **14**, 1013.

FANG, S., ANDERSON, K.M. & LIAO, S. (1969). Receptor proteins for androgens. On the role of specific proteins in selective retention of 17-beta-hydroxy-5-alpha-androstan-3-one by rat ventral prostate *in vivo* and *in vitro*. *J. Biol. Chem.*, **244**, 6584.

FAWCETT, D.W. (1973). The blood : testis barrier. In *Handbook of Physiology, Vol. 5, Section 7: Male Reproductive System*, Hamilton, D.W. & Greep, R.O. (eds) p.21. American Physiological Society: Washington, DC.

FURR, B.J.A. (1988). Pharmacological uses and potential clinical utility of ICI 176,334: a novel, non-steroidal, peripherally-selective antiandrogen. In *Hormonal Therapy of Prostatic Disease: Basic and Clinical Aspects*, Motta, M. & Serio, M. (eds) p.148. Medicom Europe: Netherlands.

FURR, B.J.A., VALCACCIA, B., CURRY, B., WOODBURN, J.R., CHESTERTON, G. & TUCKER, H. (1987). ICI 176,334: a novel non-steroidal, peripherally-selective antiandrogen, *J. Endocrinol.*, **113**, R7.

HEDLUND, P.O., GUSTAFSSON, H. & SJOGREN, S. (1980). Cardiovascular complications to treatment of prostatic cancer with estramustine phosphate (Estracyt) or conventional estrogen. A follow-up of 212 randomised patients. *Scand. J. Urol. Nephrol. (Suppl.)*, **55**, 103.

HENRIKSSEN, P. & EDHAG, O. (1986). Orchidectomy versus oestrogen for prostatic cancer: cardiovascular effects. *Br. Med. J.*, **293**, 413.

HUGGINS, C. & HODGES, C.V. (1941). Studies on prostatic cancer; effect of castration, of estrogen and of androgen injection on serum phosphatases in metastatic carcinoma of the prostate. *Cancer Res.*, **1**, 293.

HUGGINS, C., STEPHENS, J.E. & HODGES, C.V. (1941). Studies on prostatic cancer; effects of castration on advanced carcinoma of prostate gland. *Arch. Surg.*, **43**, 209.

JACOBI, G.H., ALTWEIN, J.E., KURTH, J.K.H., BASTING, R. & HOHENFELLNER, R. (1980). Treatment of advanced prostatic cancer with parenteral cyproterone acetate: a phase III randomised trial. *Br. J. Urol.*, **52**, 208.

MAINWARING, W.I.P., FREEMAN, S.N. & HARPER, B. (1987). Pharmacology of antiandrogens. In *Pharmacology and Clinical Uses of Inhibitors of Hormone Secretion and Action*, Furr, B.J.A. & Wakeling, A.E. (eds) p.106. Balliere-Tindall: Oxford.

MARKIEWITZ, M., VENEEMA, R.J., FINGERHUT, B., NEHME-HAILY, D. & SOMMERS, S.C. (1969). Cyproterone acetate (SH714) effect on histology and nucleic acid synthesis in the testes of patients with prostatic carcinoma. *Invest. Urol.*, **6**, 638.

MURTHY, L.R., JOHNSON, M.P., ROWLEY, D.R., YOUNG, C.Y.F., SCARDINO, P.T. & TINDALL, D.J. (1986). Characterization of steroid receptors in human prostate using mibolerone. *Prostate*, **8**, 241.

NERI, R.O. (1977). Studies on the biology and mechanism of action of nonsteroidal antiandrogens. In *Androgens and Antiandrogens*, Martini, L. & Motta, M. (eds) p.179. Raven Press: New York.

NERI, R.O., FLORANCE, K., KOZIOL, P. & VAN CLEAVE, S. (1972). A biological profile of a nonsteroid antiandrogen SCH 13521 (4′-nitro-3′-trifluoromethylisobutyranilide). *Endocrinology*, **91**, 427.

NERI, R.O., & MONAHAN, M. (1972). Effects of a novel non-steroidal antiandrogen on canine prostatic hyperplasia. *Invest. Urol.*, **10**, 123.

NEUMANN, F., GRAF, K.-J., HASAN, S.H., SCHENCK, B. & STEINBECK, H. (1977). Central actions of antiandrogens. In *Androgens and Antiandrogens*, Martini, L. & Motta, M. (eds) p.163. Raven Press: New York.

NEUMANN, F. & STEINBECK, H. (1974). Antiandrogens. In *Handbook of Experimental Pharmacology, Vol. 35, Section 2: Androgens and Antiandrogens*, Eichler, O., Farah, A., Herken, H. & Welch, A.D. (eds) p.235. Springer-Verlag: Berlin.

RAYNAUD, J.-P., BONNE, C., BOUTON, M.-M., LAGRACE, L. & LABRIE, F. (1979). Action of an non-steroid antiandrogen, RU23908, in peripheral and central tissues. *J. Steroid Biochem.*, **11**, 93.

RAYNAUD, J.-P., BONNE, C., MOGUILEWSKY, M., LEFEBVRE, F.A., BELANGER, A. & LABRIE, F. (1984). The pure antiandrogen RU23908 (Anandron), a candidate of choice for the combined antihormonal treatment of prostatic cancer patients: a review. *Prostate*, **5**, 299.

SAGALOWSKY, I.A. (1985). Endocrine therapy for prostate cancer. *Spec. Top. Endocrinol. Metab.*, **7**, 101.

SCATCHARD, G. (1949). The attraction of proteins for small molecules and ions. *Ann. NY Acad. Sci.*, **51**, 660.

SCHANKER, L.S. (1965). Passage of drugs into and out of the central nervous system. *Antimicrob. Agents Chemother.*, **5**, 1044.

SCHILLING, K. & LIAO, S. (1984). The use of radioactive 7-alpha, 17-alpha-dimethyl-19-nortestosterone (Mibolerone) in the assay of androgen receptors. *Prostate*, **5**, 581.

SCOTT, W.W. & SCHRIMER, H.K.A. (1966). A new oral progestational steroid effective in treating prostatic cancer. *Trans. Am. Assoc. Genito-urin. Surg.*, **52**, 95.

SMITH, R.B., WALSH, P.C. & GOODWIN, W.E. (1973). Cyproterone acetate in the treatment of advanced carcinoma of the prostate. *J. Urol.*, **110**, 106.

SODERSTEN, P., GRAY, G., DAMASSA, D.A., SMITH, E.R. & DAVIDSON, J.M. (1975). Effects of a non-steroidal antiandrogen on sexual behavior and pituitary-gonadal function in the male rat. *Endocrinology*, **97**, 1468.

TRAISH, A.M., MULLER, E. & WOTIZ, H.H. (1986). Binding of 7-alpha, 17-alpha-dimethyl-19-nortestosterone (mibolerone) to androgen and progesterone receptors in human and animal tissues. *Endocinology*, **118**, 1327.

VETERANS ADMINISTRATION CO-OPERATIVE UROLOGICAL RESEARCH GROUP (VACURG) (1967). Carcinoma of the prostate: treatment comparisons. *J. Urol.*, **98**, 516.

WEIN, A.J. & MURPHY, J.J. (1973). Experience in the treatment of prostatic carcinoma with cyproterone acetate. *J. Urol.*, **109**, 68.

WILLIAMS, G. (1985). Endocrine treatment of prostatic cancer. *J. R. Soc. Med.*, **78**, 797.

WILSON, E.M. & FRENCH, F.S. (1976). Binding properties of androgen receptors. Evidence for identical receptors in rat testis, epididymis and prostate. *J. Biol. Chem.*, **251**, 5620.